# Supplementary figures and images for: Deciphering the molecular mechanisms of mother-to-egg immune protection in the mealworm beetle Tenebrio molitor
Source: PLoS Pathog. 2020 Oct 15;16(10):e1008935. doi: 10.1371/journal.ppat.1008935 (PMC7591081; doi:10.1371/journal.ppat.1008935)

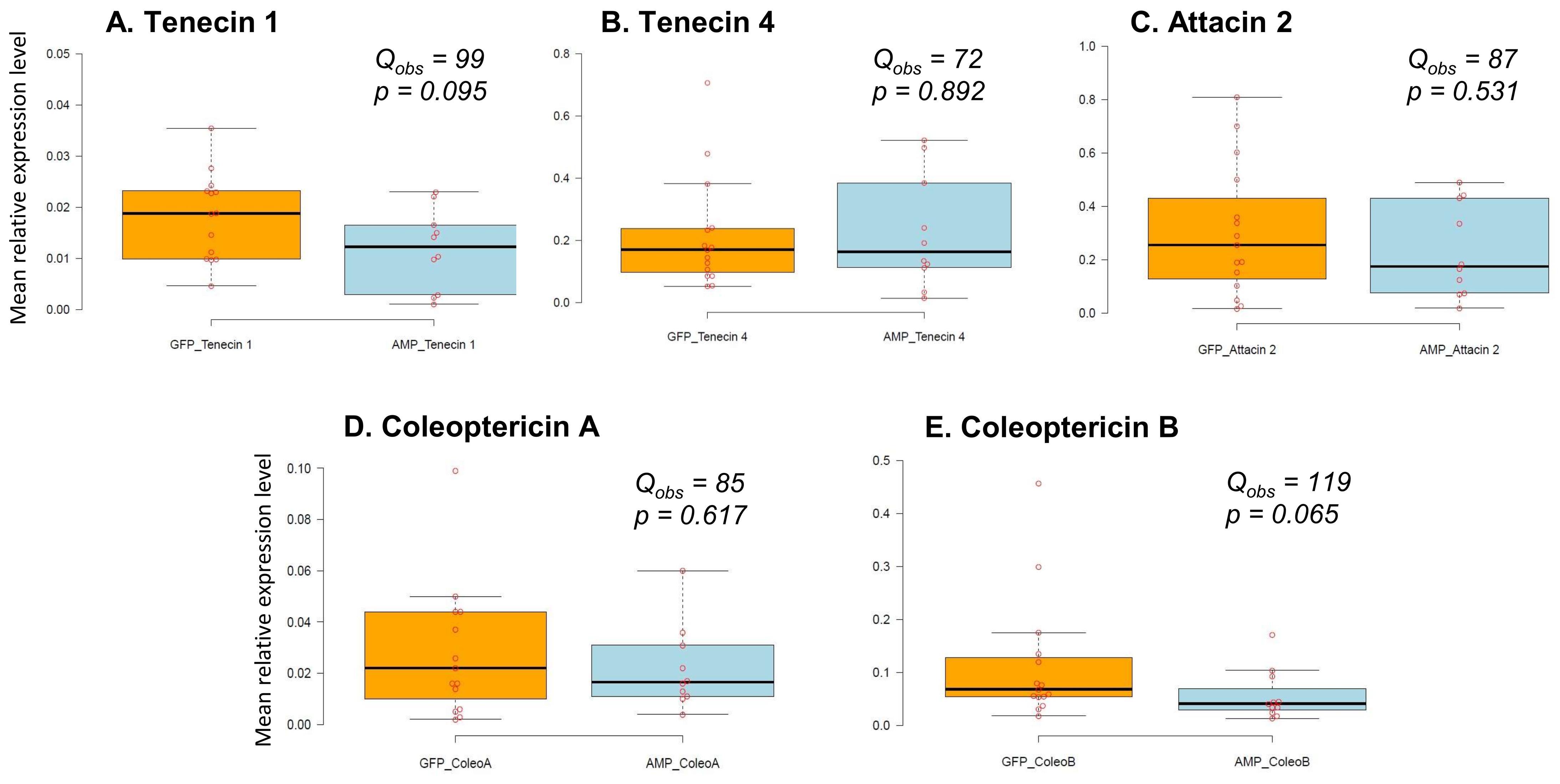

Supplement: S1 Fig — The box plots represent the mean relative expression compared to housekeeping genes (18S ribosomal RNA and Ribosomal protein L27a) for the 5 AMPs tested (tenecin 1 (A), tenecin 4 (B), attacin 2 (C), coleoptericin A (D) and coleoptericin B (E)) following treatment with GFP dsRNA (orange) or with dsAMP (blue). Results of the Mann Whitney test are indicated above box plot for each gene tested, showing no significant reduction of AMP gene expression due to high inter-individual variations following dsRNA injections. (TIF) [file ppat.1008935.s001.tif]
