# Supplementary material for: Deciphering the molecular mechanisms of mother-to-egg immune protection in the mealworm beetle Tenebrio molitor
Source: PLoS Pathog. 2020 Oct 15;16(10):e1008935. doi: 10.1371/journal.ppat.1008935 (PMC7591081; doi:10.1371/journal.ppat.1008935)
Supplement: S7 Table — (DOCX) [file ppat.1008935.s008.docx]

| **Gene name** | **GenBank_ID/ Transcript_ID** | **Primer sequence (5’-3’)** | **Amplicon size (pb)** |
| --- | --- | --- | --- |
| 18S | X07801.1/ | F: ACTGTCGGCGTACAAAC  R: CAGAACATCTAAGGGCATCAC | 97 |
| 28S | U65184.1/ | F: CGTTCTCATTGCGAGAAG  R: TTCGTCCTGACCAGGCATA | 82 |
| RPl 27a | X99204.1/  c101000_g3_i2 | F: CGGTAATGCTGGTGGTATGC  R AACTGGAGCTTTGCCTTCAG | 205 |
| RPl 13a | /  c83807_g1_i3 | F: GCGTCATTTGTCTGAAACCT  R: AGCCTTAACTTTACGTTTAGACTC | 119 |
| Tenecin 1 | D17670.1/ | F: GGAAGCTAAAGGTGTTAAACTCA  R: TCTTTCCGTTACAGTAACCTCC | 89 |
| Tenecin 2 | KF957600.1/  c101868_g1_i1 | F: ACACTACGAATTTGACCCCGA  R: TTGCCCCTTTCATCTCTCCC | 177 |
| Tenecin 3 | U21482.1/ | F: CTTGATTCTGGTGGTCGC  R: CCTTGGTGACCGGTTTG | 81 |
| Tenecin 4 | AB669089.1/ | F: CGGTGGGAAGCTGGATTACA  R: TGGACGATGCACCACCATAC | 179 |
| Attacin 2 | MF754108.1/  c94080_g1_i1 | F: TCAACAACGGAGACCATCGT  R:CTCCACAGGTTCGCATTTCC | 193 |
| Coleoptericin A | KF957599.1/  c102099_g2_i1 | F: ACGATTTCAATGCAGGGT  R: TCTTTCATCCCAACTCCTCT | 109 |
| Cecropin | /  c101352_g1_i1 | F: TCCATATCAATATCCAGGATACCC  R: AAAGGTCTTCGATTCCGTT | 92 |
| Transferin | /  c100022_g3_i1 | F: CTGAACGATAAGACCGTACCTAAA  R: TTTGAAAGGCGGTCCATAAT | 111 |
| Perilipin | /  CL8008Contig1 | F: CGACACTCAAGTAAGCTCAG  R: GATTCGACCACGGGAAG | 81 |
| Pro-Phenol-Oxydase | AB020738.1 | F: AGGAGAGGCGAGTTGTTCTA  R: GTCGTTGAACCTGGTGG | 102 |
| Vitellogenin 103138 | AY714212.1/  c103138_g1_i1 | F: CGGTCTCTGGCAAACCTA  R: CATATCCCACCACTCCTG | 99 |
| Vitellogenin 105348 | AY714212.1/  c105348_g2_i1 | F: CAAGGCGACGTTAAAGCTCA  R: TTCAAATTCTGCGTGGACC | 87 |
| Vitellogenin CL3720 | AY714212.1/  CL3720Contig1 | F: CAGGAACACGGTGGGTAA  R: AGTGTAGAGTTGTTCGGC | 94 |
| Alpha-1-Tubulin | /  c98755_g2_i1 | F ATCCCGCACCTCAAGTA  R: ACCATGAAGGCGCAATC | 97 |
