# Supplementary material for: Deciphering the molecular mechanisms of mother-to-egg immune protection in the mealworm beetle Tenebrio molitor
Source: PLoS Pathog. 2020 Oct 15;16(10):e1008935. doi: 10.1371/journal.ppat.1008935 (PMC7591081; doi:10.1371/journal.ppat.1008935)
Supplement: Attachment — Submitted filename: Response To Reviewers_V3.docx [file ppat.1008935.s009.docx]

**Editor general comment**

Both reviewers agree that this is an important and interesting topic that is eminently suitable for PLoS Pathogens. However, they have have raised important concerns. In particular, the lack of any immune priming effect in Figure 3B undermines the central conclusion of the manuscript. It seems clear that this could only be addressed using new data. This would also provide the opportunity to address some of their other suggestions about this experiment. For example, using younger eggs before translation of embryonic AMPs could occur would make the interpretation of these results more clear cut. As this likely requires a major experiment to be repeated it is unclear to me whether this work could be completed within the timeframe of a major revision (this could be discussed with the journal staff).

**General response**

We are grateful to both reviewers and to the editor for considering our work as important and suitable for PLoS Pathogens and for helping us improve this work with constructive comments. We understand the concerns and we did our best to address all of them (replies to specific comments in the text below). As both reviewers and editor raised two major concerns regarding the RNA interference experiment, we wish to address this question as a whole below to improve clarity.

1. Regarding “the lack of any immune priming effect in Figure 3B”, thanks to the comment of reviewer 1, we realized that the legend of the figure 3 contained an important error, and we apologize for that. As mentioned in the material & methods section and in the result & discussion section, eggs of the control sample originated from primed (and not from unprimed) females. This has been corrected in the legend of the figure 3, and modifications have also been made in the text to make sure this point is absolutely clarified. Our results therefore show that i) eggs from primed, AMP-dsRNA injected mothers are less likely to display antibacterial activity than the eggs of primed, GFP-dsRNA injected mothers, and that ii) the eggs of primed, GFP-dsRNA injected mothers are as likely to be protected as eggs from primed, PBS-injected mothers. We hope that with this clarification “the lack of any immune priming” is not a concern anymore. Please, see the responses to reviewer 1 for our specific response to the suggestion to use younger eggs.
2. Regarding the need to validate RNAi efficiency at the transcript level, we agree with both reviewers but we must acknowledge the long and tedious journey of the RNAi investigation. The rationale of the simultaneous use of a cocktail of multiple dsRNAs targeting five different candidate AMPs as presented in the manuscript arose from many preliminary investigations of dsRNA efficacy. We invested extensive efforts to optimize and validate RNAi experiments. In addition to the classical tests using various dsRNA concentrations and injection periods, we tested the effect of dsRNA from single AMPs (as suggested by the reviewer 2), a single exposure to dsRNA (instead of 2) and also compared two delivery methods: injection and ingestion (which is supposedly less traumatic and should therefore reduce variability). Unfortunately, results were always hampered by an important variability in AMP expression after exposure to dsRNA (see figure provided at the end of this letter). Although expression of AMPs always seemed to be lower with dsAMP than with dsGFP, the high variability in their expression prevented any statistical test to support the difference. This was not associated with a difference in phenotype. We interpreted it as a failure of dsRNA to significantly reduce expression of AMP and to provide a detectable phenotype.

This prompted us to mix dsRNAs targeting all 5 candidate AMPs genes simultaneously to investigate whether this could strengthen the potential effect on the phenotype. Although important variations in AMP expression still hampered validation of RNAi (see figure provided at the end of this letter), we observed that the number of eggs protected per female decreased, while the zone of inhibition of protected eggs remained similar to PBS and dsGFP-treated bacteria-primed females (Figure 3). We are convinced that the results and the conclusions drawn from them are solid. Indeed, AMPs gene silencing can be limited (i.e., differentially affecting the target AMPs and/or only partially reducing the AMPs expression) and have some off-target activity, increasing expression of other AMPs or triggering AMP-related pathways. But, even in this worst-case scenario, we would only underestimate the effect of AMPs on the TGIP phenotype observed. This is why we were very cautious in avoiding to specifically point to one particular AMP as a major candidate in the manuscript and we rather highlighted “AMPs” in general as important in TGIP, opening the way to further candidate gene approach to validate their specific involvement in TGIP.

In summary, given the numerous trials to improve RNAi results, we do not believe that additional RNAi experiments may help improving our mechanistic understanding with regards to the scenario 3. However, we think that the effect detected on the number of protected eggs, observed after injection of a cocktail of AMPs dsRNA, is biologically relevant and that the results still stand and deserve to be presented in the results. Therefore, to address the reviewers concerns as well as possible, we substantially modified the text as follow:

We acknowledge the high expression variability upon dsRNA treatment and clarified the rationale of the choice of the dsAMP cocktail in the Material and Methods section (pages 31, lines 714-725):

*“Despite extensive investigation efforts, important individual variations in AMPs expression levels prevented the observation of a firm effect of injection of a single AMP ds-RNA on the under-expression of AMPs in mothers. Efforts to reduce variability included using non-intrusive delivery method of dsRNA ingestion [60,61], testing the effect of a single or double exposure to dsRNA and testing several time points after exposure to dsRNA. None of these trials resulted in decreased variations in AMP expression levels in mothers and in a significant effect on eggs antimicrobial activity. Of note, it has been shown in Drosophila model that the knock-down of single TEP genes with redundant functions was insufficient to significantly impact the insect phenotype and that a change in microbial infection resistance was only observed upon the knock-down of all 4 TEP genes simultaneously [82]. Therefore, we decided to inject females with a cocktail of different candidate AMP genes to maximize the potential phenotype effect on the eggs.”*

We also clarified the procedure that we used while describing the results (pages 15-16, lines 336-351):

*“To further investigate the direct transmission of antimicrobial immune effectors into eggs from primed mothers, we conducted a RNA interference approach aimed at comparing the antimicrobial protection of 3-4 days old eggs laid 8-days post-priming of females that were injected 1-day post-priming with either i) PBS used as control of priming efficiency, ii) dsRNA from GFP as a control for the effect of non-relevant dsRNA, or iii) a cocktail of dsRNA from various AMPs (Tenecin 1 (Defensin), Tenecin 2 (Coleoptericin B), Coleoptericin A, Tenecin 4 (Attacin) and Attacin 2). In this experiment, eggs did not show significant differences in antimicrobial activity, when measured as the size of the zone of inhibition (Fig 3-A), but showed a significant difference when the proportion of eggs exhibiting an antimicrobial activity was measured. This proportion of protected eggs significantly decreased from 36.5% in eggs recovered from PBS-injected primed females to 10.3% in eggs originated from dsAMP-injected primed females (Fisher exact test, p = 0.012) (Fig 3-B). The latter matches the basal level of protection (~7-8%) of 3-days old eggs laid by PBS-injected unprimed mothers previously reported in [19]. In the same time, the injection of dsGFP had no effect on the proportion of eggs protected (Fisher exact test, p = 0.819), highlighting that the observed effect is not procedural but biologically relevant (Fig 3-B).”*

We also thoroughly discussed the limitation of our approach in regard with the obtained results, including the possibility of off-target effects pointed by reviewer 2 (pages 16-17, lines 352-370):

*“Important individual variations were observed during all RT-qPCR performed to monitor AMP candidate gene expression in RNAi-treated mothers. Considerable variations in response to dsRNA has previously been reported in insects, including coleopteran, and has been linked to multiple contributing factors including dsRNA instability/degradation, delivery method, dsRNA uptake efficiency, etc. [60,61]. In addition to a variation in RNAi efficiency (i.e. variation in silencing effect), it is possible that dsRNA (which mimics a viral infection) induces a variable non-specific immune response involving an increase in AMP expression. This would be consistent with the fact that we observed substantial variations in AMP expression in both females exposed to AMP ds-RNA and exposed to non-relevant GFP dsRNA. This would be consistent with the variation in AMP expression observed in females exposed to non-relevant GFP dsRNA. Despite this variability in AMP expression upon dsRNA treatment, our results show that eggs from primed GFP-dsRNA injected mothers are as likely to be protected as eggs from primed PBS-injected mothers, while eggs from primed AMP-dsRNA injected mothers are significantly less likely to display antibacterial activity. Therefore, even in the worst-case scenario where silencing of AMPs is limited (i.e., differentially affecting the target AMPs and/or only partially reducing the AMPs expression), we would only underestimate the effect of AMPs on the TGIP phenotype observed, even if we cannot discriminate the effect of each specific AMP independently. These results hence provide evidence that AMPs are actively transferred as effectors by mothers into the eggs, providing efficient protection of a fraction of mature eggs (3-4 days old) laid by mothers 8 days after their immune priming.”*

**Reviewer's Responses to Questions**

**Part I – Summary**

Reviewer #1: In this manuscript by Tetreau et. al., the authors describe four distinct scenarios which could contribute to trans-generational immune priming (TGIP) against bacterial pathogens in invertebrates: (1) transfer of a “signal” to eggs which could influence gene expression in eggs, (2) transfer of mRNA to eggs, (3) transfer of antibacterial effectors to eggs, and (4) epigenetic modifications. The authors employ a variety of approaches to determine which scenario(s) contribute to TGIP at the egg stage in the eggs of Tenebrio molitor females exposed to Bacillus thuringiensis and/or Serratia entomophila. Based on their data, the authors rule out the possibility that scenarios 1 or 2 substantially contribute to TGIP. In contrast, they suggest that direct transfer of effectors such as antimicrobial peptides does play a role in TGIP (scenario 3). Scenario 4 was not investigated in the present study.
While the authors convincingly demonstrate that scenarios 1 and 2 are unlikely to substantially contribute to TGIP with respect to the 11 genes under consideration, it should be made clear that their results are in fact limited to these 11 genes and should not be interpreted as an indication that scenarios 1 and 2 do not contribute to TGIP as a whole. The results in support of scenario 3 are less compelling. While the results do indeed suggest that AMPs are more abundant in the eggs of primed mothers when the eggs are laid more than three days post-priming (table 2) and that maternally deposited AMPs condition the number of eggs exhibiting antibacterial activity in 3-4 day old eggs when they are laid 4-5 days post-priming (Fig. 3b), it is difficult to link AMP deposition in the eggs, priming of mothers, and TGIP without more appropriate controls. In fact, that the eggs laid by dsGFP-injected, primed mothers were not more likely to exhibit antibacterial activity than the eggs of uninjected, unprimed mothers suggests that TGIP was not even observed in this experiment. The results presented in Fig. 5 are consistent with previous observations of TGIP and support the authors’ conclusions with regard to scenarios 1 and 2, but offer little in the way of mechanistic understanding with regards to scenario 3 given the shortcomings of the experiment depicted in Fig. 3.

Reviewer #2: The manuscript by Tetreau et al. addresses the interesting and important question what the mechanisms underpinning trans-generational immune priming (TGIP) could be. The phenomenon of TGIP describes the enhanced protection of offspring from parents that were immune primed, and has been shown in a number of (mostly) insect species, including the organism that was used here, the mealworm beetle T. molitor. Previous studies that also partly addressed the question of mechanisms of TGIP in general, and in this species in particular, are rather incomplete and give an inconsistent picture. It was not clear whether epigenetic processes are involved, or immune eliciting components (e.g. bacteria-derived substances) are transferred to eggs and/or offspring (leading to stronger activation of egg/offspring immune responses), or whether immune effectors are directly provided into the eggs. The present manuscript uses a large set of experiments and in particular proteomic approaches, showing that the latter possibility is the most relevant for this system. This is the first time, that such a complete data set and combination of experiments was used to address this question. It should be noted, that it is hard to fully exclude the other possibilities as (additional) mediators of TGIP, and in particular for other species. Also, only maternal TGIP can be explained with these mechanisms, not paternal TGIP, a phenomenon also shown for this and other species. However, even in light of these limitations, the strength of the data and the completeness of the study for this system, which is the probably most important model at the moment for maternal TGIP, makes this study very valuable.

We thank the two reviewers for their support and constructive comments.

The major concern raised here by reviewer 1 has been addressed in the “general response” above, being an unfortunate mistake in the legend of the Figure 3 that was misleading; we apologize for that.

We agree with reviewer 2 that this study is restricted to the maternal effect and we further clarified it in the “author summary” (page 3, line 67: *“characterization of maternal TGIP mechanisms in a model insect, the mealworm beetle Tenebrio molitor”*), in the introduction (page 4, lines 90-92: *“While maternal transgenerational protection has been extensively studied [6], evidence of paternal offspring immune protection has also been provided [23,24], although the underlying mechanisms might differ”*) and in the result section (page 7, line 154: *“Maternal TGIP was studied in the mealworm beetle Tenebrio molitor”*).

**Part II – Major Issues: Key Experiments Required for Acceptance**

Reviewer #1: In addition to the general concerns described above, the authors must address the following major concerns:

Figure 3: The authors should indicate the threshold zone of inhibition required to classify an egg as exhibiting antibacterial activity. For example, all eggs with zone of inhibition > 0 mm or all eggs with zone of inhibition > 1 mm. See also Fig. 5.

An egg was considered protected as soon as an inhibition zone was visible on the petri dish (Figure 3-B), irrespective of its size (qualitative observation). The size corresponds to the zone of inhibition (quantitative observation) (Figure 3-A).

Figure 3: The results presented in this figure do not support the hypothesis that priming-influenced maternal deposition of AMPs in the eggs conditions the number of eggs exhibiting antibacterial activity. The eggs of the primed, dsAMP injected mothers were less likely to display antibacterial activity than the eggs of primed, dsGFP injected mothers, suggesting that AMPs deposited by the mother do indeed influence antibacterial activity in the eggs. However, it is difficult to link antibacterial activity in the eggs to priming of the mother because the eggs of primed, dsGFP injected mothers were not more likely to display antibacterial activity than the eggs of unprimed, uninjected mothers. Useful controls for this experiment would be to also use eggs from unprimed, dsAMP injected mothers and from unprimed, dsGFP injected mothers. This would allow one to more directly evaluate the effects of priming.

We understand the reviewer concern that originates from an error in the legend of the Figure 3, which we described in more details in the general reply above. Since the purpose of this experiment was not to further investigate the effect of priming (investigated in the Figures 2, 4 and 5) but rather to investigate the effect of RNAi on primed females, and since the apparent “lack of immune-priming” has been clarified, we hope that repeating the experiment with additional controls is not a compulsory requirement anymore.

This experiment could also be greatly improved by testing the antibacterial activity in eggs on the same day they are laid. The interpretation of the present results is muddied by the developmental processes occurring in the eggs during the 3-4 day maturation period. If TGIP really is due to maternal deposition of AMPs, then this should be evident in eggs at the moment they are laid.

The post-priming days were chosen based on a previous study (Zanchi *et al.*, 2012), as already stated page 18 lines 391-397 and page 20 lines 443-445, to match the days post-priming at which the eggs are the most protected, to maximize our chances to observe a phenotype. Concerning the choice of the eggs age, we agree that studying freshly laid eggs would have been good to investigate the effect of direct effector transfer	as well. Indeed, analyzing RNAi results on 3-4 days old eggs would at worst lead to an attenuated phenotype, but would not compromise the conclusion driven on these RNAi results. The increased antibacterial protection of freshly laid eggs upon mother priming has already been reported in a previous study (Dhinaut *et al*., 2018). We therefore added a sentence in the results & discussion section to highlight the consistency of our hypothesis of direct transfer of effectors by mothers in freshly laid eggs in regards with the results published in Dhinaut *et al.*, 2018. The sentence page 20, lines 447-449 now reads: *“However, the egg protection observed herein matches with the high expression level of candidate genes in 5 days-primed females, suggesting that females might directly transfer effectors to their eggs, protecting them as early as 1 day after they are laid until 7 days post-laying [19].”*

Additionally, the authors should consider the possibility that reducing the expression of AMPs in the mothers may limit the ability of the mothers to respond to the infection and properly promote TGIP in the eggs. Thus, it is possible that the observed reduction in the number of eggs exhibiting antibacterial activity in the dsAMP treatment may only be indirectly related to the levels of AMPs in the mothers.

Yes, we also believe that this is what happens in mothers following injection of dsAMPs. Eggs protection by mothers should be the result of a secondary effect linked to the activation of mother immunity to fight the infection. When mothers were infected, they expressed AMPs that participate to self-protection, but some of these AMPs could be accumulated in eggs and then enhance egg protection in a TGIP process. Thus, when we reduce the expression of AMPs using dsRNA, we reduce the ability for mothers to properly respond to the infection and then to efficiently protect all their eggs, resulting in a reduction of the number of eggs exhibiting antibacterial activity.

Finally, the authors should present data indicating the efficiency of gene knockdown in the dsAMP vs. dsGFP mothers.

We agree with the reviewer. We provide a detailed response to this concern in the general reply (see above).

Line 455-457: “Our RT-qPCR results demonstrated that the level of immune gene transcripts in young eggs (1 and 3 days old) was too low to significantly affect the amount of effectors and sustain antibacterial activity in eggs.” This is not demonstrated by the data. The RT-qPCR data demonstrates that the expression of immune genes was below the detection threshold in ~90% of 1 day old eggs, below the detection threshold in ~70% of 3 day old eggs, and near the detection threshold in 1 and 3 day old eggs in which expression could be detected. The expression levels that would be required to “significantly affect the amount of effectors” and how these levels relate to the detection thresholds of the RT-qPCR assays are not discussed.

We agree that the link between the transcript level and the amount of effectors could not be made based on the presented results on 1d and 3d old eggs. The sentence has been modified (page 21, lines 482-486) and now reads: *“We demonstrated that the level of immune gene transcripts in young eggs (1 and 3 days old) was too low to be detected by RT-qPCR approach. Considering that the RT-qPCR is a highly sensitive method generally able to detect as low as ~10-50 transcript copies per µg of RNA [66,67], this could suggest that the low expression level in young eggs might not be sufficient to significantly affect the amount of effectors.”*

Lines 478-: “considering that 7-days old eggs from 1-day primed females did not exhibit an increase in antibacterial activity…” There is a problem with the wording here. The authors do not report increases or decreases in antibacterial activity in the eggs. Instead they report the proportion of mothers laying eggs with antibacterial activity.

This was indeed a bad wording here that could have been misleading. The sentence has been modified (page 22, lines 510-511) and now reads: *“[…] considering that the proportion of 7-days old eggs from 1-day primed females exhibiting an antibacterial activity did not increase.”*

Lines 539-540: It’s not clear how the deduced protein sequences were obtained. FrameDP is a translation tool that could be used to translate predicted coding sequences in the transcriptome, but Uniprot is a website that provides access to various tools and databases. How exactly was Uniprot used in this process?

Our transcriptome was translated using FrameDP (version 1.2.0, Gouzy *et al*., 2009). We used the program with default parameters, with for instance peptides between 300 to 500 aa. This tool uses a protein database for machine learning to determine the genetic code of our species. The reference proteome database we used for its learning phase was Uniprot (version 29/04/2015). The use of Uniprot has been clarified in the M&M section.

Line 672: It is stated here that control females were injected with PBS. This seems to contradict line 1005, which states that control females were not injected.

Reviewer 1 here correctly points the discrepancy between the legend of the Figure 3, which contained a mistake, and the material & methods section, which correctly described the experimental procedure. This is now fixed.

Lines 717-718: This information is missing from table S6. There is an RT-qPCR page in the excel spreadsheet, but it is blank.

This is now fixed.

Figure 5: The authors should define what is meant by “protected eggs”. Is this eggs with any zone of bacterial inhibition?

As stated above for Figure 3, an egg is considered protected when an inhibition zone is visible on the petri dish, irrespective of its size.

Reviewer #2: It is somewhat surprising that the transcriptomic approach was not used for assessing gene expression differences in primed mothers and eggs. Rather, it seems that the transcriptome that was constructed here was only used to assist protein identification in the proteomic data. It would be straightforward to make use of the basis that was provided here to also explore more deeply the gene expression differences following priming. Instead, gene expression differences where here studied with a rather limited approach, focusing on a few candidate genes (Fig. 4). The results obtained by this approach are interesting and support the conclusions drawn from the proteomic approach, but it would be good to obtain a clearer picture that could e.g. hint why AMPs were down-regulated in eggs at day 5 (which is somewhat surprising). However, given that the proteomic data already provides a lot of information, I probably wouldn't say that this additional experiment is absolutely required, but it is still surprising not find any such data in the manuscript.

The transcriptomic reference database was indeed constructed to allow the proper identification of protein candidates, which would be strongly limited in the absence of a reliable database for the species studied. This was stated in the first paragraph of the “results & discussion”, and repeated elsewhere in the “R & D” and “M & M” sections. The transcriptome, as it was prepared, cannot be directly exploited to explore gene expression differences, as it is made of a mix of 150 individuals at different developmental stages, sex and physiological conditions. It is true that in the absence of a genome, it can be used to explore differences in transcriptomes (e.g. used to map the reads) and we made it available to the scientific community for that purpose. This statement was added page 9, lines 195-198 to further justify the relevance of the transcriptome in the present study.

We agree with the reviewer that RNAseq data are always more comprehensive than RT-qPCR on a selected set of candidate genes and that in an ideal context, the choice of the former should prevail on the latter. Considering the complexity of the experimental priming set-up and the number of conditions tested combined with the limited funds available for the project, we had to make a choice; an increase in transcriptomic depth of analysis by RNAseq would have resulted in a reduced number of experimental time-point sampling. Exploring the differences in transcriptome after priming in both the mothers and eggs at various time points (as in the experiment of the Figure 4) represents a substantial amount of work (that would require 6 transcriptomes x minimum 3 replicates). We preferred to address the question of the scenario 3 by two global proteomic approaches to identify candidates and investigate the scenario 1 and 2 by RT-qPCR on a selected subset of candidate genes.

The RNAi approach lacks confirmation that it really leads to knock-down of the target genes. A cocktail of 5 dsRNAs were used here, and I am a bit sceptical that all these genes were down-regulated? Using so many genes might also increase the risk of off target effects. More information, in particular RT-qPCR data is needed here.

We agree with the reviewer. We provide a detailed response to this concern in the general reply (see above).

**Part III – Minor Issues: Editorial and Data Presentation Modifications**

Reviewer #1: I also have some minor concerns the authors may wish to address:
The manuscript is generally clearly written, but throughout the manuscript text and figures the authors should take care to consistently note the age of eggs and the number of days post-priming at which the eggs were laid. The reader is forced to frequently consult the methods section and figure 6 to interpret many of the results and understand the experimental setup. For example, none of this information is provided in Fig. 3 or the figure legend for this figure.

It is true that the sampling set-up is complex but it was necessary to properly investigate the TGIP phenomenon and disentangle the different scenarios. The egg ages indeed differ from one experiment to another and it can be hard to follow, even with the summary Figure 6. In accordance with the reviewer’s comment, we added the egg ages in several places in the manuscript (page 10 line 207, page 13 lines 293-294, page 15 line 338, page 18 lines 400-401, page 27 lines 613-615, page 29 lines 670-671), in the title of Table 3 and in the legends of Figures 2 and 3.

Line 174: I suggest the authors use the ExN50 value, rather than the N50 value, as an assessment of the quality of their de novo transcriptome assembly. The N50 value is more appropriate for genome assemblies and can be a misleading statistic for transcriptome assemblies.

We agree with the reviewer that the newly developed metrics ExN50 is more accurate than N50 for transcriptomes. The corresponding values have been added to the Table 1 for our transcriptome. We nevertheless also keep the N50 value to be comparable to the other published coleopteran transcriptomes for which only N50 values are available.

Table 2: With the exception of catalase in the control>bacteria row, no proteins are seen as significantly differentially abundant in both eggs laid and eggs in ovaries. Is this to be expected?

This has been indeed a surprising result. As stated page 10, lines 204-218, we believe that the higher number of proteins observed in the eggs collected from ovaries could come from contamination with nurse cells, although special care was taken to avoid sampling them. Results from eggs freshly laid are therefore more representative of the effectors eventually present in the egg to protect it, explaining why we only focused on these eggs for the LC-MS/MS complementary global proteomic approach.

Lines 518-523: The use of insects of various developmental stages and physiological conditions is reasonable, but the authors should be more specific in their description of the insects used to create the sequencing libraries. How many males and females? How many insects from each developmental stage? How many bacterially or phenobarbital challenged individuals were used and at what timepoint post challenge were they collected?

The condition of individuals (stage, treatment, etc.) used for the library construction was added in the M&M section page 24, lines 551-560:

*“Because we aimed at generating a database enriched in transcripts involved in immune and stress responses, the sequencing was performed on pooled RNA from individuals of various developmental stages (third instar larvae, pupae, adults), sex (males and females) and physiological conditions. More precisely, groups of 10 individuals of third instar larvae, adult males and adult females, were either not treated or injected with B. thuringiensis, or with S. entomophila bacteria as described above, or injected with the drug phenobarbital (0.1%), known to induce a detoxification response in insects [69]. We also used RNA from 30 pupae (untreated because of their higher sensitivity to stress), resulting in a total of 150 individuals. RNA was extracted 48h after priming, using trizol method (TRIzol LS Reagent, Invitrogen) according to the manufacturer's instructions.”*

Lines 527 and 532: I suggest the authors specify that these read counts refer to paired-end reads (i.e. the total read count is twice the stated number). This would facilitate comparison to table 1, where total read count is given.

This has been specified in the text (page 25, line 564).

Line 532: The authors state that they sequenced two libraries and after quality control, these libraries contained 58 and 44 million reads (102 million reads). Based on table 1 it seems that these reads were pooled into one set of reads for de novo assembly? If so, this should be explicitly stated.

The two libraries were indeed pooled and this is now clearly stated in the text (page 25, line 569).

Line 539: “Traduction” seems to be a French word. Do the authors mean “translation”? See also line 615.

Line 539: “FrameDB” should be “FrameDP”.

These are both corrected in the new version of the manuscript (page 8, line 179, page 25, lines 577-579 and page 28, line 654).

Line 555-567: For the 2-D-DiGE experiment, the authors state that laid eggs were collected within 16 hours following oviposition and that collection began after 3 days post-priming. Given that the number of days post-priming at which an egg is laid seems to have important implications for the antibacterial potential of the egg, it would be very useful for the reader if table S6 included a column indicating the number of days post-priming at which each egg was collected. Additionally, it is not clear in the manuscript text or in table S6 at which day(s) post-priming eggs in ovaries were collected. Similar information should be provided for eggs collected for nano-LC-MS/MS (lines 631-635).

We agree that such information should be added in more places in the manuscript for facilitating the reading and improving the clarity. All eggs for proteomics (2D-DiGE and LC-MS/MS) were collected 3 days post priming. We accordingly added this information in several places in the manuscript (page 10 line 207, page 13 line 295, page 27 line 614-615, page 29 line 670-671), in the title of Table 3 and in the legend of the Figure 2 and of the Table S6.

Line 1010: The authors should indicate the statistical test used. On line 339 it is stated they a used a Fisher’s exact test, but this should be restated in the figure legend.

This is now stated in the legend of Figure 3. We added the statistical test used for Figure 5 as well.

Line 686: I suggest the authors refer specifically to the dsRNAs injected rather than “respective knocking down treatments”.

This has been clarified in the text page 32, lines 740-742:

*“On day 4, females were injected again with either i) PBS as control for injection procedure, ii) GFP-dsRNA (0.6 µg/µL) as control for non-relevant dsRNA, or iii) a cocktail of dsRNA from tenecins 1 and 4, coleoptericins a and b, and attacin-2 […]”*

Line 690-693: The description of the egg collection scheme is difficult to understand. As far as I can tell, eggs were laid on days 8 and 9 (corresponding to 4 and 5 days post immune priming, respectively) and then allowed to mature until day 11, at which point the eggs were either 3 or 4 days old. This is all there in the manuscript, but not conveyed in the clearest manner.

As stated in response to the comments above, all these informations have now been clarified throughout the manuscript (information on egg ages, time post-priming, etc.) and we hope that this is now much clearer despite the obvious complexity of the RNAi experimental procedure.

Line 730: There is a typo (plateform vs. platform).

This is now corrected.

Reviewer #2: Summary: It generally captures very well the content of this study, just a detail:
- In my view, the wording is sometimes a bit exaggerated. For example, I would not call this a 'global multidisciplinary approach'.

We agree with the reviewer and the sentence now reads: *“This work exemplifies how combining comparative global approaches helps to disentangle the different scenarios of a complex trait”*

A similar sentence present at the end of the introduction has also been changed accordingly (page 7, lines 147-148): *“The different scenarios of such a complex trait could be disentangled by a combination of global comparative approaches”*.

Introduction: It captures the literature and open questions very well.
Results & Discussion: This section generally reads well, but might be shortened to make it more consistent. I only have a few suggestions:

- Make clear that the intention of producing the transcriptomic data was here only for assisting the proteomic approach. Because of this, the part on the transcriptomic data could also be shortened. I am not sure whether Table 1 needs to be in the main paper, maybe something for the supplement?

We clarified our intention to build a reference transcriptomic database for assisting the proteomic approach in the present study as stated in previous comments. However, because this database is available to the scientific community and, we hope, will be useful to future proteomic and transcriptomic studies, we feel it is important to provide the typical database information. This database will not be published elsewhere and the present manuscript will be the reference for all future database users. We would therefore prefer maintaining the amount of information and keeping the Table 1 in the main paper to improve its visibility to future users.

- I suggest somewhat more general explanations of the proteomic techniques that were used here, because some readers will not be specialists for these techniques. What is e.g. meant with 'top-down proteomics analysis of eggs extracts' (l. 291-2)

Considering the complexity of the study and experimental design, we chose to not include too many technical details in the Results and Discussion. We believe that the high level of details that we provided in the M&M is sufficient to address this reviewer’s concern and justify the choice and relevance of the different methods.

- It is not always clear how many samples or replicates were used. Please add this important information also to the R&D section (not only the methods part).

Information on the number of replicates and/or total number of individuals used have been added page 8 lines 173-174, page 13 line 295, page 32 line 750, and in the title of Figure 2 and Figure 5.

- In the last section (l. 423 ff.) it should be made clearer that the literature is sometimes on data obtained in other species. For example, Knorr et al (2005) was on Tribolium castaneum, i.e. even though these are both coleopterans, still other mechanisms might be relevant in this species than in Tenebrio molitor. Other data was even obtained in honey bees, etc. Moreover, the mechanisms relevant here cannot be relevant for paternal TGIP. In summary, it cannot be excluded that different species employ different mechanisms for TGIP, and this should be more clearly stated. This also relates to the Abstract: is T. molitor really a 'model' for clarifying TGIP mechanisms (on a general level), or do we need to be very modest and it is only maternal TGIP to eggs in T. molitor where the mechanism could be clarified here? In my view, this doesn't make the data less valuable.

The last part of the “results & discussion” section was indeed not specifying the species on which each of the results discussed was obtained. This has been clarified in the new version in several places (page 21 line 477, page 22 lines 494, 496-499, 501-502, page 23 line 515). We thank the reviewer for this comment as this has made this part much more precise and accurate.

Materials and Methods:

- l. 497: please provide information about the B.t. strain. Was it a B.t. tenebrionis? Which Cry toxins does it have? It is not straightforward to get this information via the strain number CIP...

We voluntarily did not want to indicate the *Bt* subspecies as we believe it is not a relevant information in the context of this article, which aims at comparing two natural pathogens that are here considered as representative of Gram positive or Gram negative bacteria. We are worried that adding such information would be more misleading than informative, considering that the subspecies information is not available for *Serratia entomophila*. Moreover, the bacteria are used for priming during their vegetative phase, and therefore, no Cry toxin is present in the priming bacterial mixture as they are produced only during the *Bt* sporulation phase.

- l. 501 and other places: I suggest using the term 'priming' when inactivated pathogens are used, and challenge for life pathogens (which were not used here). It makes more sense given the whole phenomenon is termed TGIP.

“Priming” has replaced the word “challenge” for consistency throughout the manuscript according to reviewer’s comment.

- provide more details on the statistical analyses

Additional information have been added in the Material & Method section.

Page 35-36, lines 831-843:

*“Statistical analyses*

*Size of egg zones of inhibition following to RNA interference treatments were analyzed using Shapiro-Wilk test for normality and Student T-test to test differences in zone of inhibition size (p<0.05), whereas the analysis of the proportion of eggs exhibiting antibacterial activity according to RNA interference treatments were analyzed using Fisher exact tests (p<0.05). Presence of antibacterial activity among the eggs laid by control, PBS-, B. thuringiensis- and S. entomophila-treated females after 1 day or 5 days post-priming were analyzed using binomial logistic regressions.*

*The relative expression of the 11 candidate immune genes measured in adult primed females with B. thuringiensis and S. entomophila and in their 7 days-old eggs laid after 1, 5 and 12 days post-priming relative to PBS-injected control females were analyzed using Mann-Whitney tests (p<0.05), following normality verification using Shapiro-Wilk test. Analyses were made using IBM® SPSS® Statistics 19 software.”*

Figures:
Figure 3: make clear that 3A does not show values with zero (below detection) inhibition

This information has been added in the Figure 3-A legend: *“Eggs that did not exhibit antimicrobial activity (null zone of inhibition) were not considered”*

Fig. 5: show statistical analysis

This information has been added in the legend of Figure 5 (and Figure 3 as well).

Supplementary files:
Table S6: the tab 'RT-qPCR' seems to be empty

This is now fixed.

**Figure: Representative examples of variations in AMPs expression levels in primed adult females exposed to dsRNA.**

A. The three graphs show individual expression ratios of AMPs in primed adult females exposed to non-relevant GFP dsRNA (ds-GFP mothers) or to ds-RNA targeting candidate AMPs (ds-AMP mothers); here tenecin 4, attacin 2 and tenecin 2. Expression ratios are expressed relative to expression of 2 internal reference genes.
B. The Box plot represent the mean expression for the 5 AMPs tested following exposition to GFP dsRNA or to a ds-RNA for tenecin 1, coleoptericin B, coleoptericin A, tenecin 4 and attacin 2.

Global consideration:

Similar variations in AMPs expression ratios were observed regardless of the dsRNA delivery method (injection or ingestion), dsRNA nature (dsRNA from a single or multiple AMPs) or time period after the first exposure to dsRNA (5 or 8 days post-exposure).
