# Supplementary material for: Deciphering the molecular mechanisms of mother-to-egg immune protection in the mealworm beetle Tenebrio molitor
Source: PLoS Pathog. 2020 Oct 15;16(10):e1008935. doi: 10.1371/journal.ppat.1008935 (PMC7591081; doi:10.1371/journal.ppat.1008935)
Supplement: Attachment — Submitted filename: Response_V2.docx [file ppat.1008935.s010.docx]

**Editor general comment**

The reviewers are satisfied with all but one of the responses. One reviewer felt that the failure to validate the knock-down experiments by qPCR meant no useful conclusions could be drawn from this data. I note the other reviewer raised this issue in their initial review and was satisfied with the response. Having read your covering letter, I have sympathy with the frustrations of this experiment. I also agree that the GFP dsRNA control coupled with a rather specific assay for AMP activity provides an indication that RNAi is affecting AMP expression in some way. At a minimum, I would like to see the box plots presented in the covering letter and an accompanying statistical analysis as a supplementary figure. This will make explicit the issues wich are described in the results. Furthermore, in the final Discussion section this issue should be mentioned again, and it should be made clear to what extent the major conclusions depend on this result. However, the ideal validation of knock-down efficiency is to measure protein rather than mRNA levels. Given that AMP concentration can be measured using inhibition zone assays, is there any reason not to check that the treatment reduced AMP concentration in the mothers in this way? This appears to me to be a way to bypass the frustrations of the qPCR.

Dear editor,

We would like to thank you for providing us with an opportunity to address the last remaining concern on the manuscript. We are glad that all other responses and substantial modifications that we made in the previously revised manuscript properly addressed the comments from both reviewers. We are really grateful for your support, for acknowledging our efforts invested in the RNAi experiment and for sharing our frustrations with the results that were obviously not as comprehensive as we expected. Your suggestion to validate the AMP inhibition by measuring the anti-microbial activity in mothers’ hemolymph is indeed a sound alternative to the RT-qPCR verification in this context. Unfortunately, we did not think of this solution at the time of the experiment as we embraced the “classical” RT-qPCR validation strategy and all the RNAi-treated mothers have been used for RNA extraction. Considering that we do not have any of these samples available in the -80°C anymore, providing such control would mean running again the entire experiment – not only on females but also on eggs from RNAi-treated mothers to take into account potential inter-experiment variations. For several reasons involving the current sanitary situation limiting our access to the different labs combined with the end of the funding devoted to this project (and the associated manpower), we must admit that we are unable to run such a complex and tedious experiment again in the forthcoming year(s). Please be ensured that we explored all the possibilities to experimentally address this question to the best of our capacity.

We still believe that our results are valid – showing a measurable phenotype upon RNAi treatments – and that, at worst, this phenotype is attenuated but not flawed. Therefore, we added the boxplots submitted in the cover letter in the previous manuscript version as a new Figure S1 as you suggested, which is now cited page 16, line 352 in support of the following sentence: “Important individual variations were observed during all RT-qPCR performed to monitor AMP candidate gene expression in RNAi-treated mothers”.

We also clarified the absence of validation of AMP inhibition in mothers in two different places in the “results & discussion” section:

Page 17, lines 366-370: “Although we could not confirm AMP invalidation by RT-qPCR in RNAi-treated mothers and we did not investigate the antimicrobial activity in mothers’ hemolymph, these results still provide support for an active transfer of AMPs as effectors by mothers into the eggs, enabling an efficient protection of a fraction of mature eggs (3-4 days old) laid by mothers 8 days after their immune priming.”

Page 21, lines 471-476: “One should be careful that the importance of AMP involvement in TGIP could even be stronger than observed in the present study for *T. molitor*, as we might have observed an attenuated phenotype given our inability to validate the knockdown of the AMPs in mothers. This nevertheless supports that mothers actively protect eggs in a binary way: either providing them with a sufficient amount of effectors to efficiently protect them against early bacterial aggressors, or not providing any protection, leading to no-to-low antibacterial activity.”

We hope that these clarifications meet your expectations and are sufficient to avoid misleading the reader by being as transparent as possible about the strengths and weaknesses of our study. Of course, we are willing to further modify the text if you feel some parts of the manuscript still require extensive edition regarding this aspect.

There are a number of minor comments to address. In addition it seems you may not have seen the comment on data availability on the first draft:

"It seems that the authors haven't seem my previous comment regarding public access to data: For the transcriptome data, I wasn't sure whether the availability through the laboratory website is sufficient? Maybe it should also be put on one of the commonly used repositories?"
I noticed that there was a number looking somewhat like an SRA accession number on the subission form. The sequencing data either needs to be submitted to the SRA, or, if this has been done, it needs to be made clear.

In the previous version of the manuscript, the BioProject (ID PRJNA646689) was already provided, which encompass the SRA numbers. As requested, we further added the SRA numbers (SRR12235350 and SRR12235349) in the new version (page 26, lines 590-591).

It is PLoS policy to avoid repeated rounds of revisions. I therefore apologise that I have recommended a 'Major Revision'. However, this provides an opportunity to do more experiments to validate if these are feasible, and it is likely a final decision will not require further peer review.If the experiment I suggested is either impractical or inappropriate, then I can rapidly reach a decision on a revised manuscript.

Thank you again for giving us the opportunity to address the remaining points. We hope that you will find our answers to the concerns and the modifications that we made sufficient for our manuscript to be suitable for publication in PLoS Pathogens.

**Reviewer's Responses to Questions**

**Part I - Summary**

Reviewer #1: My most significant concern with the original manuscript was with regard to the experiment depicted in Figure 3. Here the authors note that the legend for this figure contained an error which has been corrected in the revised manuscript. As a result, I no longer have concerns regarding the existence of immune priming in the experiment depicted by this figure.

Reviewer #2: With the current revision, the authors have improved the manuscript. They could also clarify several issues in their response letter. No additional experiments were done, but the arguments against doing so are overall convincing. I have a few remaining, minor points regarding the authors' responses and wording in the summary of the manuscript.

We thank the reviewers for finding that our responses and modifications in the manuscript overall convincingly addressed the points that they raised.

**Part II – Major Issues: Key Experiments Required for Acceptance**

Reviewer #1: The authors have appropriately addressed all of my other concerns with the exception of the need to validate their knockdown of AMP transcripts by RNAi. The authors state that they were unable to validate the knockdown due to high inter-individual variation in AMP expression. From the figure included in the response to reviewers, it is indeed clear that there is very high variation in the expression of AMPs between individuals. Based on this figure, it seems that many dsGFP- and dsAMP-injected individuals have highly reduced AMP expression ratios and there is no discernable difference in AMP expression between dsGFP- and dsAMP-injected adults. The authors state that “even in the worst-case scenario where the silencing of AMPs is limited, we would only underestimate the effect of AMPs on the TGIP phenotype..”. Indeed a change to the TGIP phenotype is observed in the dsAMP-injected mothers compared to the dsGFP-injected mothers, but without proper validation of the AMP knockdowns, it is not appropriate to link this phenotype to reduced expression of AMPs. In the context of such high variation in AMP expression and the use of a relatively small number of mothers for this experiment, the need to validate the knockdown is particularly important.

We agree with the reviewer that this validation should ideally be provided. Please read the response to the editor above for a detailed response.

Reviewer #2: N/A

**Part III – Minor Issues: Editorial and Data Presentation Modifications**

Reviewer #1: (No Response)

Reviewer #2: Summary:

Only minor changes were made to the summary upon my criticism that the wording in the summary is in some places a bit exaggerated. However, I am afraid that I am not fully happy with these changes either. The word 'comparative' might indicate to some readers that this would be a study based on comparative approaches (i.e. species comparisons), which is clearly not the case here.
Moreover, the sentence 'present data clearly reject the involvement of mRNA' is, in my view, too strong, given the limitations of the approach that could only address mRNA for some candidate genes (I suggest deleting 'clearly').

We accordingly modified the abstract to tone down some sentences that the reviewer found exaggerated. Two sentences have therefore been modified and now read: “By taking the mealworm beetle *Tenebrio molitor* as a biological model, our results suggest that eggs are mainly protected by an active direct transfer of a restricted number of immune proteins and of antimicrobial peptides. In contrast, the present data do not support the involvement of mRNA transfer while the transmission of a “signal”, if it happens, is marginal and only occurs within 24h after maternal exposure to bacteria.”

In addition, the term “comparative” has been erased to comply with reviewer suggestion.

Authors' general response #(1):

While the clarification helps understanding the data in Fig 3 (and conclusions drawn from it), it should be noted that a comparison of primed with unprimed mothers is missing; ie. the full design would include priming and RNAi as factors. I suggest to add this consideration to the paper.

The reviewer is correct when raising the point that unprimed mothers in this experiment are absent. However, they are not missing since our purpose here was to test whether it was possible to invalidate antibacterial activity in eggs of primed mothers with a cocktail of dsRNA of all the candidate genes. We have previously shown that eggs produced by unprimed mothers do not have antibacterial activity (Zanchi et al. 2012, Dhinaut et al. 2018). We therefore felt that trying to repress antibacterial activity in eggs that generally do not express antibacterial activity was unnecessary. This is the reason why this assay did not involve eggs of unprimed mothers. With due respect to the reviewer, we do not believe that such a consideration would be of help in the paper.

Authors' general response #(2):

I appreciate the detailed discussion of the issues with validation of the RNAi using a cocktail of dsRNAs. I still consider these methodological problems a little worrying, though. The huge variability in the qPCR data used in the attempt to validate the RNAi effect could be a methodological problem (low repeatability of the assay) or large inter-individual variation (e.g. because the beetle population is diverse). Any chance to discriminate between these possibilities (e.g. repeatability of sample replicates)?

We agree that, in the worst cases, a high variability may also result from technical problems. To test this possibility, we assessed the repeatability of the RT-qPCR for replicated measures data from the genes investigated following the procedure described by Lessells & Boag, 1987 (DOI: 10.2307/4087240). The results are provided below:

|  | **Tenecin 4** | **Attacin 2** | **Coleoptericin A** | **Coleoptericin B** | **18S** | **Rpl27a** |
| --- | --- | --- | --- | --- | --- | --- |
| **n** | 30.00 | 30.00 | 29.00 | 30.00 | 30.00 | 30.00 |
| **k** | 3.00 | 3.00 | 3.00 | 3.00 | 3.00 | 3.00 |
| **Σn_i_** | 90.00 | 90.00 | 87.00 | 90.00 | 90.00 | 90.00 |
| **Σn^2^_i_** | 270.00 | 270.00 | 261.00 | 270.00 | 270.00 | 270.00 |
| **n_0_** | 3.00 | 3.00 | 3.00 | 3.00 | 3.00 | 3.00 |
| **MS_A_** | 4.97 | 16.59 | 8.70 | 5.06 | 3.55 | 1.32 |
| **MS_W_** | 0.06 | 0.16 | 0.06 | 0.02 | 0.08 | 0.03 |
| **S^2^** | **0.06** | **0.16** | **0.06** | **0.02** | **0.08** | **0.03** |
| **S^2^_A_** | **1.63** | **5.48** | **2.88** | **1.68** | **1.16** | **0.43** |
| **r** | **0.96** | **0.97** | **0.98** | **0.99** | **0.93** | **0.94** |

**n**: group sample

**k**: number of replicated measures

**Σn_i_**: sample size across groups and replicates

**Σn^2^_i_**: sample size across groups and squared replicates

**MS_A_**: Mean square among groups from the analysis of variance (see table below)

**MS_W_**: Mean square within groups from the analysis of variance (see table below)

**S^2^**: within group variance

**S^2^_A_**: between group variance

**r**: repeatability coefficient

Analysis of variance on gene expression measured by RTqPCR over 3 replicates per individual for the calculation of repeatability:

|  |  | SS | df | MS | F | P |
| --- | --- | --- | --- | --- | --- | --- |
| Tenecin 4 | Between Groups | 144.01 | 29.00 | 4.97 | 80.66 | <0.001 |
|  | Within Groups | 3.69 | 60.00 | 0.06 |  |  |
|  | Total | 147.70 | 89.00 |  |  |  |
| Attacin 2 | Between Groups | 481.07 | 29.00 | 16.59 | 101.65 | <0.001 |
|  | Within Groups | 9.79 | 60.00 | 0.16 |  |  |
|  | Total | 490.86 | 89.00 |  |  |  |
| Coleoptericin A | Between Groups | 243.68 | 28.00 | 8.70 | 159.19 | <0.001 |
|  | Within Groups | 3.17 | 58.00 | 0.06 |  |  |
|  | Total | 246.85 | 86.00 |  |  |  |
| Coleoptericin B | Between Groups | 146.65 | 29.00 | 5.06 | 267.01 | <0.001 |
|  | Within Groups | 1.14 | 60.00 | 0.02 |  |  |
|  | Total | 147.78 | 89.00 |  |  |  |
| 18S | Between Groups | 102.14 | 29.00 | 3.52 | 42.79 | <0.001 |
|  | Within Groups | 4.94 | 60.00 | 0.08 |  |  |
|  | Total | 107.08 | 89.00 |  |  |  |
| Rpl27a | Between Groups | 38.30 | 29.00 | 1.32 | 50.84 | <0.001 |
|  | Within Groups | 1.56 | 60.00 | 0.03 |  |  |
|  | Total | 39.86 | 89.00 |  |  |  |

The method for gene expression measures shows very high repeatability (from 0.93 to 0.99) and in all cases, variation among individuals was much higher than variation of measures within individuals. Therefore, we may infer that variation in RT-qPCR data results from large inter-individual differences rather than from methodological problems.

This additional test was performed to reply to the reviewer’s question but we do not feel appropriate to include it in the manuscript, considering that such methodological validations are never shown in RT-qPCR studies.

Material and Methods, my question regarding the Bt strain used:
I am not convinced by the argument that Bt strain information was not provided because 'it is not a relevant information in the context of this article'. The methods of a paper should be as precise as possible, e.g. to enable reproduction of the study. There are many different strains of Bt, e.g Bt tenebrionis which is infective to beetles, while other strains are not (in oral infection). Priming can be specific regarding the Bt strain in both oral and septic infections, thus this information is not irrelevant.

We kindly disagree with the argument of the reviewer saying that the information provided in the manuscript do not allow the reproducibility of the study. We provided the strain codes (CIP53.1 and CIP120919) for both bacterial strains together with the reliable source (Pasteur Institute). Any readers could therefore get access to the very same bacterial strains would they like to repeat our study. We are devoted to provide as many information as possible as long as it is informative and not misleading, which would be the case by adding an information at the subspecies level for *Bacillus thuringiensis* but not for *Serratia entomophila*.
